# Antifungal Activity of (KW)*_n_* or (RW)*_n_* Peptide against *Fusarium solani* and *Fusarium oxysporum*

**DOI:** 10.3390/ijms131115042

**Published:** 2012-11-15

**Authors:** Ramamourthy Gopal, Hyungjong Na, Chang Ho Seo, Yoonkyung Park

**Affiliations:** 1 Research Center for Proteineous Materials, Chosun University, Gwangju 501-759, Korea; E-Mail: ramagopa@gmail.com; 2 Department of Biotechnology, Chosun University, Gwangju 501-759, Korea; E-Mail: naozhong@naver.com; 3 Department of Bioinformatics, Kongju National University, Kongju 314-701, Korea; E-Mail: chseo@kongju.ac.kr

**Keywords:** lysine, arginine, tryptophan, antifungal peptides, fungicidal

## Abstract

The presence of lysine (Lys) or arginine (Arg) and tryptophan (Trp) are important for the antimicrobial effects of cationic peptides. Therefore, we designed and synthesized a series of antimicrobial peptides with various numbers of Lys (or Arg) and Trp repeats [(KW and RW)*_n_*-NH_2_, where *n* equals 2, 3, 4, or 5]. Antifungal activities of these peptides increased with chain length. Light microscopy demonstrated that longer peptides (*n* = 4, 5) strongly inhibited *in vitro* growth of *Fusarium solani*, and *Fusarium oxysporum*, at 4–32 μM. Furthermore, longer peptides displayed potent fungicidal activities against a variety of agronomical important filamentous fungi, including *F. solani* and *F. oxysporum*, at their minimal inhibitory concentrations (MICs). However, RW series peptides showed slightly higher fungicidal activities than KW peptides against the two strains. Taken together, the results of this study indicate that these short peptides would be good candidates for use as synthetic or transgenic antifungal agents.

## 1. Introduction

**Figure 1:**
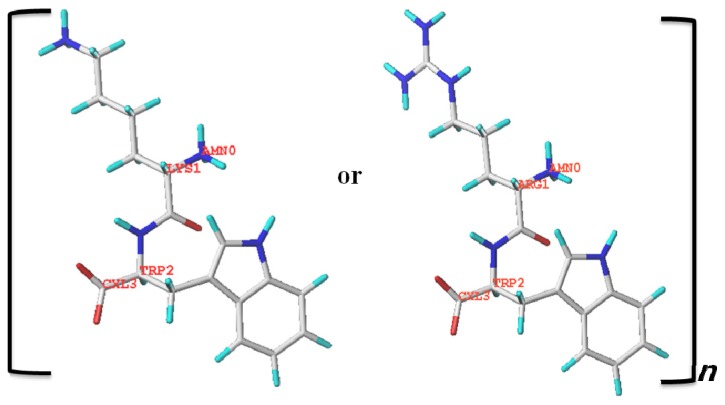
Chemical structure of linear antimicrobial peptides (AMPs) (KW)*_n_*-NH_2_ or (RW)*_n_*-NH_2_ used in this study, where *n* = 2, 3, 4, and 5.

Cationic antifungal peptides have been developed as novel biocidal agents in the battle against pathogenic microorganisms [1]. Generally, cationic peptides are 12 to 50 amino acids in length as well as amphiphilic, as they contain two to nine basic residues (arginine (Arg) or lysine (Lys)) and approximately 50% hydrophobic residues [2,3]. However, the large size of antimicrobial peptides (AMPs) hinders their use due to high manufacturing costs. Therefore, selective short AMPs have been developed based on their amino acid combinations, charge, and hydrophobicity [4–8]. In this context, AMPs containing cationic and hydrophobic amino acids constitute a promising tool to combat plant fungal pathogens. For example, both defensin and MtDef4 require cationic and hydrophobic amino acids for their antifungal activities [9]. Furthermore, this requirement has been demonstrated by the loss of antimicrobial activity upon substitution of basic residues in AMPs [10]. Other short tryptophan (Trp)-rich cationic peptides, PAF26, PAF38, PAF40, and BM0, also display antifungal activity [11,12]. The peptide indolicidin, which belongs to the cathelicidin family of AMPs, is an Arg and Trp-rich peptide with potent, broad-spectrum antimicrobial activity [13,14]. Further, a large number of cationic plant defensins exhibit inhibitory activities against filamentous fungi both *in vitro* and in transgenic plants [15–20]. It has been reported that short peptides show antifungal activity as they contain cationic and hydrophobic amino acids [21,22]. This is clearly evident based on previous reports that hexapeptides containing predominately cationic and hydrophobic amino acids show the highest antifungal activity [11,23]. Therefore, we synthesized a series of peptides containing a repeated pattern of Lys (K) or Arg (R) and Trp (W) residues, (KW)*_n_* and (RW)*_n_* (where *n* equals 2, 3, 4, or 5) (Figure 1), and determined their antifungal and fungicidal activities.

## 2. Results and Discussion

Antifungal peptides have been used in both transgenic plant and human pharmaceutical applications. Furthermore, it has been extensively reported that many short AMPs rich in Lys or Arg and Trp show antifungal activity [4,6,8,24–26]. Although it is not yet clear that these peptides kill plant fungal pathogens, it is important to optimize chain length of AMPs for the purpose of large-scale production. In the present study, we investigated the antimicrobial effects of small KW and RW series peptides against plant pathogens such as *Fusarium solani* and *Fusarium oxysporum*. Specifically, *F. solani* is a phytopathogenic fungus as well as an important causal agent of several crop diseases, including root and stem rot of pea, sudden death syndrome of soybean, foot rot of bean, and dry rot of potato [27–30]. *F. oxysporum* is a phytopathogenic fungus that affects tomato crops, causing huge losses to farmers [31].

### 2.1. Antifungal Activities against Hyphal Growth

**Figure 2:**
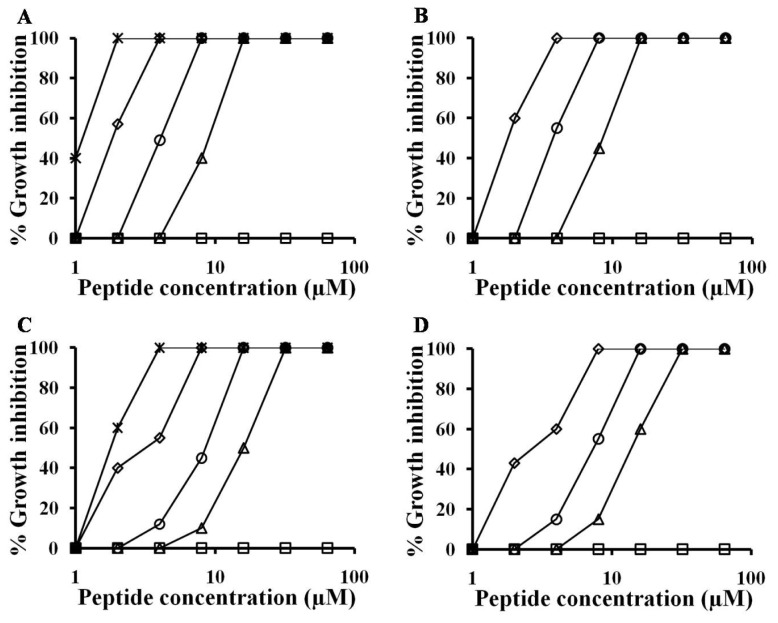
Quantitative measurement of fungal growth inhibition. (KW)*_n_*-NH_2_ (**A** and **C**) and (RW)*_n_*-NH_2_ (**B** and **D**). Antimicrobial activities of tetrapeptides (squares), hexapeptides (triangle), octapeptides (circle), decapeptides (diamonds), and melittin (cross) aganist *in vitro* growth of *Fusarium solani* (**A** and **B**) and *Fusarium oxysporum* (**C** and **D**).

**Figure 3:**
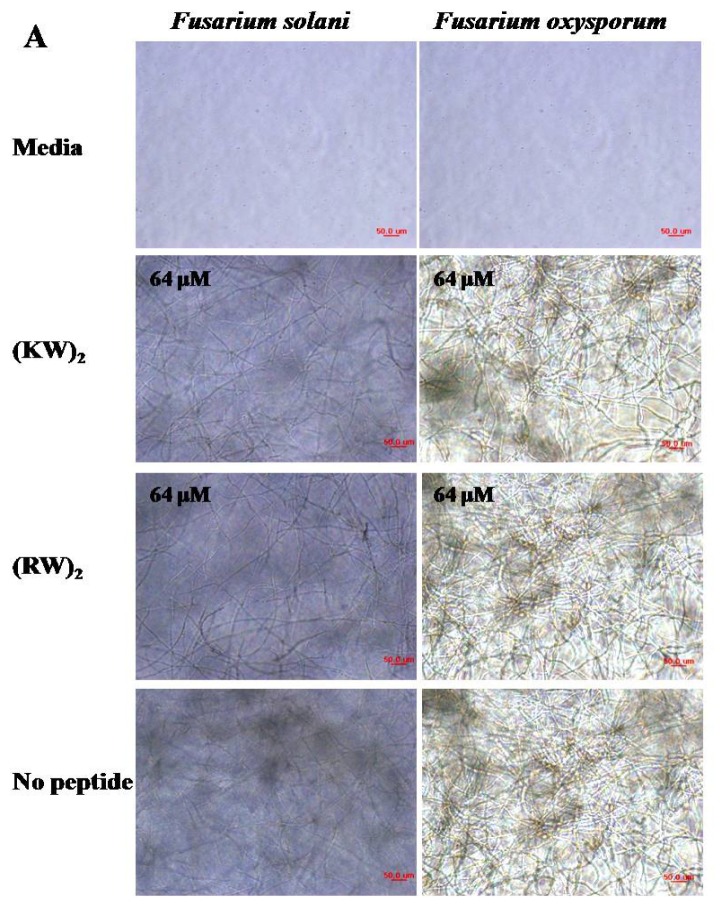

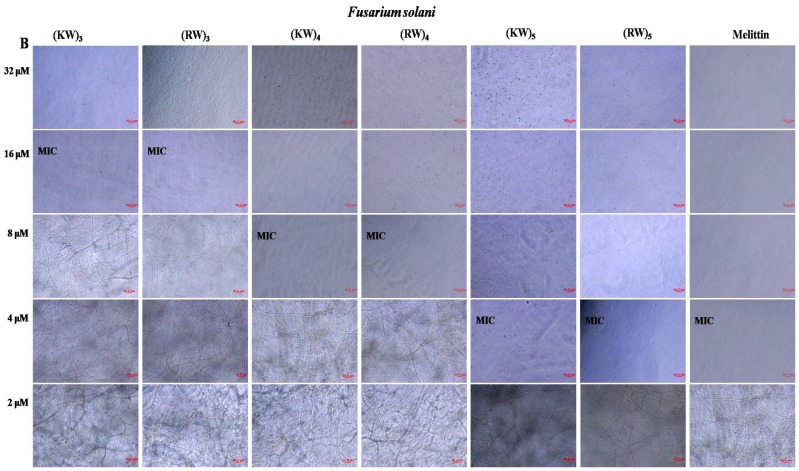

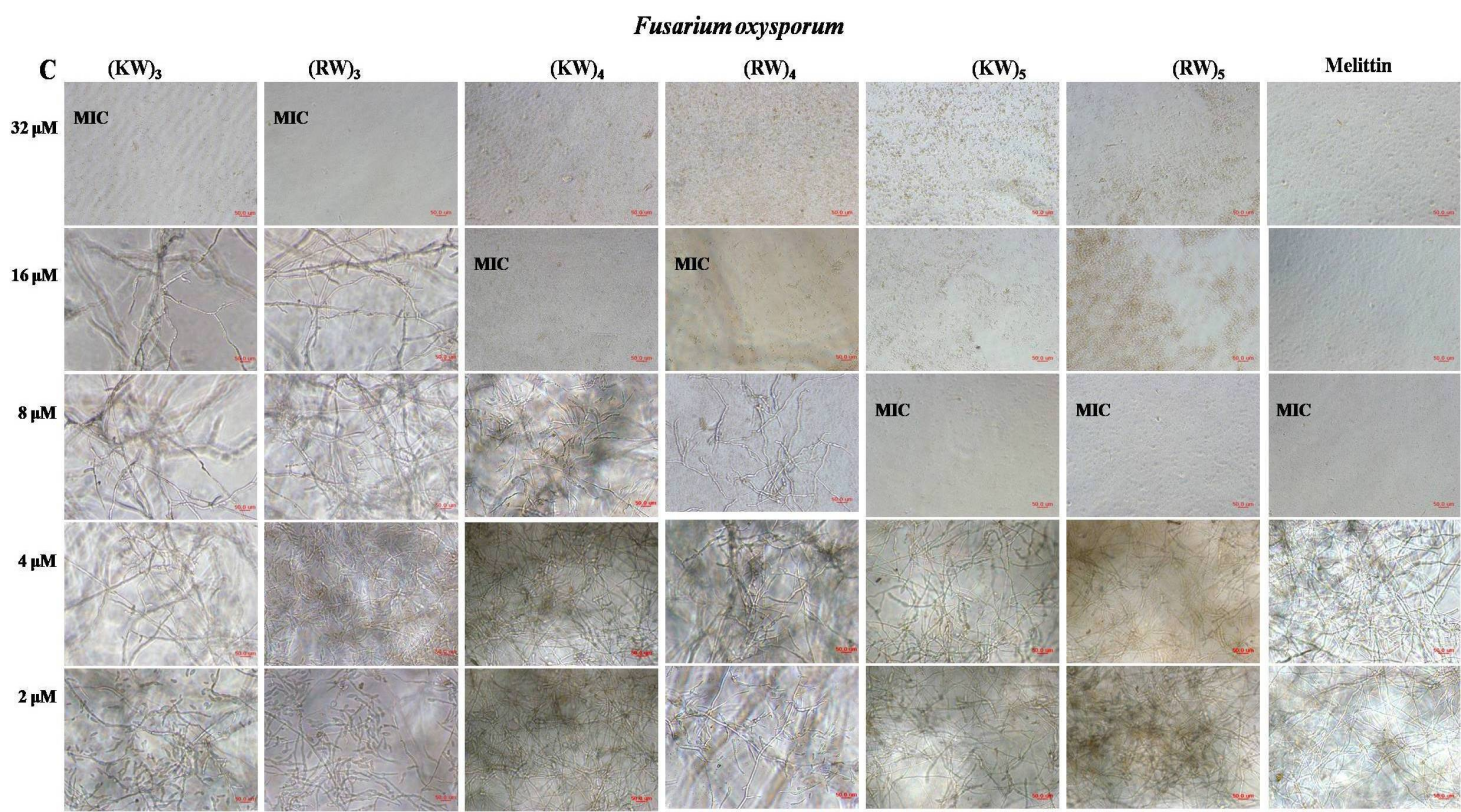
(**A**) Images showing non-inhibition of conidial germination and hyphal growth of fungal strains treated with (KW)_2_-NH_2_ or (RW)_2_-NH_2_ at 64 μM. Images showing inhibition of conidial germination and hyphal growth of *Fusarium solani* (**B**) and *Fusarium oxysporum* (**C**) at different concentrations of peptides. Bar = 50 μm.

In the present study, spectrophotometric and microscopic experiments were used to assess the antifungal activities of the peptides on hyphal growth of *F. solani* and *F. oxysporum*. As shown in Figure 2, chain length of (KW)*_n_* and (RW)*_n_* peptides strongly correlated with increasing antifungal activity. Decamer (KW and RW)_5_ peptides inhibited conidial germination completely (100% growth inhibition) at 4 μM for *F. solani* and 8 μM for *F. oxysporum*. Further, decamer peptides reduced growth rates compared to other peptides at all tested concentrations (1, 2, 4, 8, 16, 32, and 64 μM), although a concentration of 1 μM did not prevent sporulation. Similarly, (KW)_4_ and (RW)_4_ peptides exhibited antifungal effects against *F. solani* at concentrations as low as 4 μM, which is two-fold less than their minimal inhibitory concentration (MIC) (8 μM). (KW)_4_ and (RW)_4_ peptides also inhibited growth of *F. oxysporum* at concentrations below their MIC (16 μM). Specifically, at a concentration of 8 μM, (KW)_4_ and (RW)_4_ inhibited growth of *F. oxysporum* by 45% and 55%, respectively (Figure 2C,D). (KW)_3_ and (RW)_3_ peptides at a concentration of 16 μM completely inhibited germination of *F. solani* conidia. In contrast, *F. oxysporum* conidia were able to germinate and grow even in the presence of peptides at 16 μM, although 100% growth inhibition was observed at 32 μM (Figure 2C,D). Thus, (KW)_5_ and (RW)_5_ peptides exhibited significantly higher antifungal activities than other peptides and were almost as potent as melittin. Lastly, (KW)_2_ and (RW)_2_ at 64 μM did not inhibit growth of the fungal strains. The antifungal activities of peptides were in the following order: tetrameric peptides < hexameric peptides < octameric peptides < decameric peptides (Figure 2). These data suggest that a critical chain length may be required for significant antifungal activity. Melittin possesses strong antifungal activity and served as a positive control in this experiment. Photomicrographs of the mycelia of *F. solani* and *F. oxysporum* fungi were taken after a 24 h growth period. The effects of peptides on hyphal morphology were monitored and compared with the morphology of untreated hyphae (Figure 3A). Decameric and octameric peptides significantly inhibited spore germination and hyphal growth of phytopathogenic fungi in comparison with tetrameric peptides, which is consistent with the results on percentage inhibition of fungal growth. However, above 16 μM (for *F. solani*) or 32 μM (for *F. oxysporum*), (KW)_3_ and (RW)_3_ peptides noticeably inhibited conidial germination and hyphal development (Figure 3B). Significantly, hyberbranching of fungal hyphae, which is a typical morphological response of *F. solani* and *F. oxysporum* in response to (KW)_2_ and (RW)_2_, was not observed in the presence of other peptides at concentrations above or near their MICs. Therefore, we believe that antifungal activity increased with chain length. A previous study showed that (RW)*_n_* series peptides with increased chain length possess enhanced antimicrobial activity [6]. Furthermore, decameric peptides and melittin showed similar antifungal activities against *F. solani* and *F. oxysporum*. On the other hand, hexametic and octameric peptides showed decreased antifungal activities against the same two strains in comparison with melittin. This result is consistent with previous data that also indicated that small cationic hexapeptides had lower antimicrobial activities compared to longer, naturally occurring AMPs such as magainin, cecropin, and melittin [11,32]. In addition, these hexameric peptides showed higher antifungal activities than the other hexameric peptides [21], suggesting that, in this (KW)_3_ or (RW)_3_, the composition of amino acid within the repeating motif seems to be more selective than the PAF26 or PAF32 for fungal membrane.

### 2.2. Effects of Longer Peptides on Cell Viability

**Figure 4:**
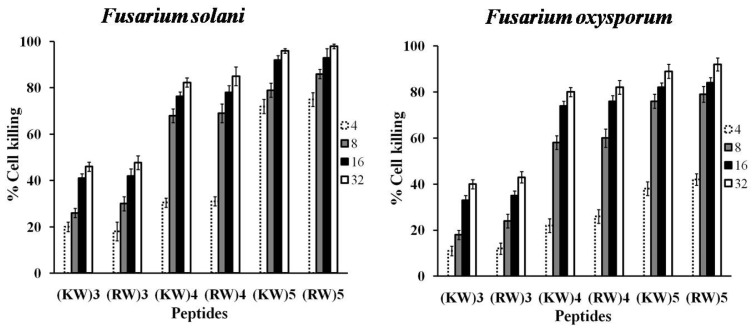
Viability of hyphal cells after treatment with antifungal peptides. Viability of fungal cells after treatment with peptides at different concentrations was monitored using an 3-(4,5-dimethylthiazol-2-yl)-2,5-diphenyltetrazolium bromide (MTT)-based assay.

To determine whether the antifungal peptides are fungicidal or fungistatic, *F. solani* and *F. oxysporum* cells were treated with or without peptides for 3 h. (KW)_3_ and (RW)_3_ peptides at their MICs showed moderate fungicidal activities against both *F. solani* and *F. oxysporum*. After treatment with (KW)_4_ and (RW)_4_ at their MICs, 68% and 69% of *F. solani* (or 74% and 76% of *F. oxysporum*) cells were killed, respectively (Figure 4). For (KW_5_) and (RW_5_) at their MICs, 72% and 75% of *F. solani* cells along with 76% and 79% of *F. oxysporum* cells were killed, respectively. The percentages for *F. oxysporum* further increased to 89% and 92% in the presence of (KW)_5_ and (RW)_5_ at 32 μM, respectively. Consistent with the results on percentage of growth inhibition, killing activity increased with peptide concentration as well as peptide length. Moreover, the results show that both (KW)*_n_* and (RW)*_n_* (*n* = 3, 4, and 5) had similar inhibitory activities against the two strains at corresponding MICs, but they differed in their fungicidal activities. In fact, RW series peptides showed slightly higher fungicidal activity than KW peptides. Some studies also reported that Arg-containing peptides were more active against plant fungal strains than Lys-containing peptides [20]. Specifically, the guanidinium group of Arg strongly interacts with fungal strains due to its higher net positive charge compared to the protonated amine of lysine. In addition, Arg-containing peptides showed a two-fold greater activity against *F. solani* than *F. oxysporum*. Taken together, our results clearly indicate that the level of antimicrobial activity depends on the peptide sequence and fungal membrane composition.

Our results also show that the peptides act as fungicidal compounds. Application of fungicidal agents is usual practice when fighting plant diseases [33]. Specifically, antifungal peptides with fungicidal activity are often used in plant transgenic applications. For example, antifungal plant defensin is induced in radish leaves upon challenge with fungal pathogens, suggesting a role in plant defense [34]. Further, the natural AMP magainin has been expressed in *Nicotiana tabacum* to develop resistance to phytopathogens [35]. Other studies also reported that transgenic plants expressing AMPs exhibit broad-spectrum resistance to phytopathogen infection [36–38]. At present, the high cost of synthetic peptides constitutes an obvious limitation to agricultural and food applications. As a result, there have been many efforts to express short AMPs in transgenic plants [38,39], for example indolicidin [40,41]. Short hexapeptides are used to control postharvest diseases in fruits and vegetables caused by fungal phytopathogens [21,23,42]. In fact, we previously identified (KW)_4_ as a potentially non-toxic AMP [43] that can be produced on a large scale economically, although this short peptide has been produced in transgenic plants. Future research will determine the feasibility of these options. Meanwhile, the modes of action of these peptides are yet to be resolved, although studies have proposed various killing mechanisms for Lys-Arg/Trp rich AMPs, including cytoplasmic membrane disruption and inhibition of nucleic acid synthesis [44]. Other studies have reported that Lys-Arg/Trp-rich AMPs kill fungal strains through nucleic acid binding or cell penetration [45,46]. Studies on the modes of action of these peptides are currently underway.

## 3. Experimental Section

### 3.1. Materials

Rink amide 4-methylbenzhydrylamine resin, fluoren-9-ylmethoxycarbonyl (Fmoc) amino acids, and other reagents for peptide synthesis were purchased from Calibiochem-Novabiochem (La Jolla, CA, USA). For the quantitative antifungal assay and fungicidal activity assay, the following fungal strains were obtained from the Korea Collection for Type Cultures (KCTC): *F. solani* (KCTC 6326) and *F. oxysporum* (KCTC 6076). Fungal cells were grown on PDA (potato dextrose agar) plate and subcultured for 2–3 weeks.

### 3.2. Peptide Synthesis and Purifications

The peptides KWKW-NH_2_ (KW)_2_, KWKWKW-NH_2_ (KW)_3_, KWKWKWKW-NH_2_ (KW)_4_, KWKWKWKWKW-NH_2_ (KW)_5_, RWRW-NH_2_ (RW)_2_, RWRWRW-NH_2_ (RW)_3_, RWRWRWRW-NH_2_ (RW)_4_, RWRWRWRWRW-NH_2_ (RW)_5_ and GIGAVLKVLTTGLPALISWIKRKRQQ (melittin) were synthesized by the solid-phase method using Fmoc chemistry on a solid support of rink amide 4-methylbenzhydrydrylamine resin. Then, 0.1 M *N*-hydroxy benzotriazole (HOBt) and 0.45 M 2-(1*H*-benzotriazole-1-yil)-1,1,3,3-tetramethyluroniumhexafluorophosphate (HBTU) in dimethylformamide (DMF) along with 2 M *N*,*N*-diisopropyl ethylamine (DIEA) in *N*-methylpyrrolidone (NMP) were used as coupling reagents, and 10-fold excess Fmoc-amino acid was added during every coupling cycle. Following a final deprotection with a solution of 20% piperidine in DMF and cleavage with a mixture of TFA/water/triisopropylsilane (90:5:5) for 2 h at room temperature [47], the crude peptides were repeatedly extracted with diethyl ether and purified using reverse phase preparative high performance liquid chromatography (HPLC) on a Vydac C_18_ column (4.6 × 250 mm, 300 Å, 5 nm). The molecular masses of the peptides were confirmed by using a matrix-assisted laser desorption ionization mass spectrometer (data not shown) (MALDI II, Kratos Analytical Ins.). The purity of all peptides were found to be > 95%.

### 3.3. Computational Modeling

Chemical structures of the peptides were built using ChemOffice Desktop 2004 for Windows (CambridgeSoft (CS) Corporation, Cambridge, MA, USA).

### 3.4. Antifungal Assay

Fungal fragments, precultured in mycelial growth medium, were placed in the center of PDA plates, after which the cultures were incubated for 96 h at 25 °C in the dark. After incubation, spores were isolated from cultures growing in half-strength PDA. Spore concentrations were then adjusted to 5 × 10^4^ spores/mL in half-strength PDA, after which 80 μL was added to the wells of sterile 96-well flat-bottomed microtiter plates along with 20 μL of peptide or media to give final concentrations of 1–64 μM. Several wells were kept untreated as a control to monitor fungal growth. Plates were incubated in the dark at 25 °C for 24 h before hyphal growth was determined by measuring optical density at 595 nm using a microtiter plate Elisa reader (Molecular Devices, Sunnyvale, CA, USA) [48]. Each test was performed in triplicate. Percentages of inhibition were then calculated (Figure 1) (0% inhibition indicates growth equal to control sample (only media)) [21]. The lowest concentration of peptide inhibiting fungal growth was monitored microscopically with an inverted light microscope (IX71, Olympus, Tokyo, Japan) [49].

### 3.5. Cell Viability Assay

The spore suspension at a concentration of 5 × 10^4^ spores/mL (80 μL) was transferred to a 96-well microtiter plate along with 20 μL of (KW)*_n_* or (RW)*_n_* peptides (*n* = 3, 4, and 5), melittin, or media to give final peptide concentrations of 4–32 μM. Plates were incubated for 3 h before the addition of 10 μL of 3-(4,5-dimethylthiazol-2-yl)-2,5-diphenyltetrazolium bromide (MTT; 5 mg/mL; Sigma). Plates were then incubated again for 16 h at room temperature, followed by the addition of 100 μL of MTT solvent (0.1 N HCl in anhydrous isopropyl alcohol). Presence of MTT/formazan was monitored spectrophotometrically by measuring the absorbance at 570 nm and then subtracting the background absorbance at 690 nm using a Versa-Max microplate Elisa reader (Molecular Devices, Sunnyvale, CA, USA) [48]. Each measurement was conducted in triplicate.

## 4. Conclusions

In summary, increased chain length along with a higher ratio between hydrophobicity and net charge resulted in increased antifungal and fungicidal activities. Our results confirm that KW and RW peptide elements could be incorporated into the development of an AMP with low cost due to their short lengths, making these peptides a promising alternative tool in the field of green biocides.
